# Incidence and outcomes of cancer treatment-related cardiomyopathy among referrals for advanced heart failure

**DOI:** 10.1186/s40959-018-0029-y

**Published:** 2018-03-20

**Authors:** Raquel Araujo-Gutierrez, Sergio H. Ibarra-Cortez, Jerry D. Estep, Arvind Bhimaraj, Ashrith Guha, Imad Hussain, Myung H. Park, Guillermo Torre-Amione, Barry H. Trachtenberg

**Affiliations:** 1 Department of Heart Failure & Transplant Cardiology, Houston Methodist DeBakey Heart & Vascular Center, Houston Methodist Research Institute, 6565 Fannin St, F657, Houston, TX 77030 USA; 2 Department of Structural Heart Disease, Houston Methodist DeBakey Heart & Vascular Center, Houston Methodist Research Institute, 6565 Fannin St. F766, Houston, TX 77030 USA; 3 Department of Heart Failure & Transplant Cardiology, Houston Methodist DeBakey Heart & Vascular Center, Houston Methodist Hospital, 6550 Fannin St. Suite 1901, Houston, TX 77030 USA

**Keywords:** Chemotherapy-induced cardiomyopathy, Anthracyclines, Heart failure, Heart transplantation, Cancer–related cardiomyopathy, Heart transplant, Mechanical circulatory support, Left ventricular assist device, Radiation-induced cardiomyopathy

## Abstract

**Background:**

Approximately 2–3% of patients undergoing advanced heart failure therapies such as left ventricular assist devices (LVAD) and orthotropic heart transplantation (OHT) have chemotherapy-related cardiomyopathy, according to analyses of large databases such as United Network for Organ Sharing (UNOS) or Interagency Registry for Mechanically Assisted Circulatory Support (INTERMACS) registries. While these studies have shown similar survival outcomes post-interventions, these databases by definition exclude patients referred for advanced therapies but do not receive them, and thus there is little data on overall outcomes of such patients. Given the lack of nuance in the diagnoses in large registries and the possibility that many cancer treatment-related cardiomyopathy (CCMP) patients might be misclassified by the generic “non-ischemic” or “dilated” cardiomyopathies, we investigated the incidence and clinical outcomes of CCMP patients among advanced heart failure (HF) referrals at a single high volume institution.

**Methods:**

All referrals from 2013 to 2016 were evaluated for type of cardiomyopathy, with careful chart review. Outcomes such as LVAD, OHT and death were compared between CCMP and other cardiomyopathies.

**Results:**

Of 553 referrals for advanced HF, 19 (3.4%) were for CCMP. There was a higher percentage of patients receiving advanced therapies in the CCMP vs. non-ischemic cardiomyopathy (NICMP) and ischemic cardiomyopathy (ICMP) (42.1% vs 30.2% vs 33.6%, not significant). Of the CCMP patients, 3 had OHT directly, 2 had LVAD followed by OHT, and 3 had LVADs as bridge to candidacy or destination therapy. Fifty-eight percent of the CCMP did not receive LVAD or OHT compared to 69.8% and 66.3 of the NICMP and ICMP, respectively (*p* = 0.0388). Independent of type of advanced therapy, survival was significantly higher in the CCMP group compared to NICMP and ICMP (93.3% vs 84.8% vs 73.8%, respectively *P* = 0.0021 for 1 year, 93.3% vs 76.2% vs 58.3%, respectively, *P* = < 0.0001 for 3 year).

**Conclusions:**

In a single institution, CCMP accounts for more than 3% of all referrals for advanced HF therapies and almost 8% of NICMP. Contrary to concerns for previous cancer and sequelae of cancer treatment excluding patients for advanced therapies, a higher percentage of CCMP underwent advanced HF therapies and with similar outcomes. This is the first study to show that among patients referred for advanced therapies, CCMP patients do not have inferior outcomes compared to other cardiomyopathies regardless of the selected management strategy.

## Background

Advances in anti-neoplastic therapies have increased the number of long-term survivors over the last decade, with 64% of patients surviving 5 years or more after diagnosis, 41% surviving 10 years or more, and 15% surviving 20 years or more [1]. However, the survival gain has not come without a cost as there is an increasing number of cancer survivors with cardiotoxic effects due to chemotherapeutic agents, radiation, or both [2]. Anthracyclines remain the most common chemotherapy agent to cause cancer treatment-related cardiomyopathy (CCMP). CCMP has been reported in up to 10% of cancer survivors, including childhood cancer survivors treated with doxorubicin, adult patients treated for Hodgkin lymphoma, and women treated for breast cancer with chemotherapy and/or radiation, progressing to end-stage heart failure (HF) in 2% to 3%, according to estimates based on retrospective registry data [3–7]. For example, according to analyses of large databases such as United Network for Organ Sharing (UNOS) or Interagency Registry for Mechanically Assisted Circulatory Support (INTERMACS) registries, approximately 0.5% to 2.5% of patients undergoing advanced heart failure therapies such as left ventricular assist devices (LVAD) and orthotropic heart transplantation (OHT) have CCMP [8–10]. While these studies have shown similar survival outcomes post-interventions compared to patients with other types of cardiomyopathy, these databases by definition exclude patients referred for advanced therapies who receive medical therapies and do not meet criteria for OHT or LVAD. Given the lack of nuance in the diagnoses in large registries and the potential for misclassification by the generic “non-ischemic”, “idiopathic” and/or “dilated” cardiomyopathies, we aimed to investigate the incidence of CCMP among advanced HF referrals at a single high-volume institution. In addition, the focus on referrals is novel and will demonstrate if there is a higher incidence with advanced HF that are declined for advanced therapies and thus not represented in preexisting registries for LVAD or heart transplant. We hypothesized that survival would be similar between patients referred for advanced therapies for patients with CCMP compared to ischemic cardiomyopathies (ICMP) and non-ischemic cardiomyopathies (NICMP).

## Methods

### Study population

All referrals from January 2013 to April 2016 were evaluated for type of cardiomyopathy through careful chart review to ensure accuracy. We analyzed patients’ demographics, and outcomes of mortality, LVAD implantation, and OHT. We compared these findings among CCMP, NICMP and ICMP patients, and we also compared patients with CCMP to patients with all other cardiomyopathies combined (O-CMP). Additionally, pre-implant and pre-transplant laboratory values, hemodynamic parameters, echocardiographic measurements and pulmonary function tests were compared between the 3 groups. Lastly, the incidence of right ventricular (RV) failure was analyzed in patients receiving LVAD as advanced therapy, and compared between the 3 groups. RV failure was defined as more than 14 days on inotropic support or the need for a right ventricular assist device (RVAD).

### Statistical analysis

Data were represented using means with standard deviation for numeric variables, and percentages and counts for categorical variables. Baseline characteristics are represented using percentages or mean with standard deviations, as applicable. Survival and composite outcome estimates were calculated using Kaplan-Meier method and compared using log-rank (Mantel-Cox) test. Categorical characteristics and clinical outcomes were compared between groups using one way ANOVA, unpaired t-test, and Fisher’s extract, as appropriate. Comparisons for continuous variables were made using Wilcoxon-Mann-Whitney test. A *p* value of < 0.05 was considered statistically significant.

## Results

### Study population

Amongst the 663 patients referred for advanced HF therapies, 110 were excluded from analysis due to incomplete or missing chart data. A total of 553 patients were included in the analysis. We retrospectively identified 19 patients with CCMP (3.4%) via chart review. Thirteen of these 19 patients (68%) were already appropriately diagnosed in history and physical records/charts, as CCMP, while 6 patients (32%) were previously diagnosed as either NICMP, “dilated cardiomyopathy” (DCM) or “idiopathic cardiomyopathy” and thus reclassified as CCMP. The diagnoses submitted to INTERMACS for CCMP patients who received LVAD as destination therapy (DT) were familial (*n* = 1), CCMP (*n* = 2); The INTERMACS and UNOS diagnoses for patients who received LVAD as bridge to transplant (BTT) were: idiopathic (*n* = 2), CCMP (*n* = 1). UNOS diagnosis for OHT patients: idiopathic (*n* = 2), CCMP (*n* = 1). CCMP diagnosis was defined as cardiomyopathy not due to coronary artery disease or another obvious secondary cause in a patient treated with any anthracycline chemotherapy and/or radiation (involving the chest cavity) prior to the onset of HF symptoms. Furthermore, 225 patients were diagnosed with NICMP (40.7%) and 309 with ICMP (55.9%). Baseline demographic characteristics for 3 groups are represented in Table 1. The CCMP cohort was predominantly female (CCMP 68.4% vs NICMP 30.7% vs ICMP 15.2%; *P* < 0.0001). Patients in the ICMP group were significantly older (mean age: CCMP 57.8 yrs. vs NICMP 50.2 yrs. vs ICMP 61.1 yrs., *P* < 0.0001).

**Table 1:** Baseline demographics and advanced heart-failure therapies

	CCMP n (%)	NICMP n (%)	ICMP n (%)	*p* Value (All groups)	*p* Value CCMP vs NICMP	*p* Value CCMP vs All other
	19 (3.4)	225 (40.7)	309 (55.9)			
Age, (yrs) Mean (SD)	57.8 ± 12.4	50.1 ± 13.9	61.1 ± 9.7	< 0.0001	0.0196	ns
Male n (%)	6 (31.6)	156 (69.3)	262 (84.8)	< 0.0001	0.0008	< 0.0001
White n (%)	9 (47.4)	76 (33.8)	153 (49.5)	0.0012	ns	ns
Black n (%)	6 (31.6)	101 (44.9)	67 (19.7)	< 0.0001	ns	ns
Hispanic n (%)	4 (21)	34 (15.1)	46 (14.9)	ns	ns	ns
Total LVAD n (%)	5 (26.3)	33 (14.7)	79 (25.6)	0.0013	ns	ns
DT	3 (15.8)	23 (10.2)	61 (19.7)	0.0029	ns	ns
BTT	2 (10.5)	10 (4.4)	18 (5.8)	ns	ns	ns
Total OHT n (%)	5 (26.3)	45 (20)	43 (13.9)	ns	ns	ns
OHT only	3 (15.8)	35 (15.5)	25 (8.1)	0.0150	ns	ns
OHT, VAD or Both n (%)	8 (42.1)	68 (30.2)	104 (33.6)	ns	ns	ns
Medical therapy *n (%)*	11 (57.9)	157 (69.8)	205 (66.3)	0.0388	ns	ns

### Clinical outcomes

#### LVAD and OHT

Clinical outcomes for all groups are represented in Table 1. Patients with CCMP and ICMP diagnosis were more likely to receive durable mechanical circulatory support (MCS) compared to NICMP patients (26.3% vs 25.6% vs 14.2%, respectively, *p = 0.0013).* However, patients with ICMP were significantly more likely to have durable LVADs as destination therapy (DT) strategy, compared to other cardiomyopathy categories (CCMP 15.8% vs NICMP 10.2% vs ICMP 19.7%; *P = 0.0029*). There was a higher percentage of patients receiving LVAD as bridge to transplant (BTT) in the CCMP group compared to O-CMP (10.5% vs 4.4% vs 5.8%, *ns*). The percentage of total transplanted patients (including BTT) was higher in the CCMP group (CCMP 26.3% vs NICMP 20% vs ICMP 13.9%; *ns*). Similarly, patients from the CCMP and NICMP significantly received more transplants (not including BBT) compared to ICMP (15.8% vs 15.5% vs 8.1%, respectively, *P = 0.0150*). Overall, more patients in the CCMP group received advanced heart failure therapies (LVAD, OHT or both) compared to the other 2 groups. (CCMP 42.1% vs NICMP 30.2% vs ICMP 33.6%, *ns*).

#### Survival

**Fig. 1:**
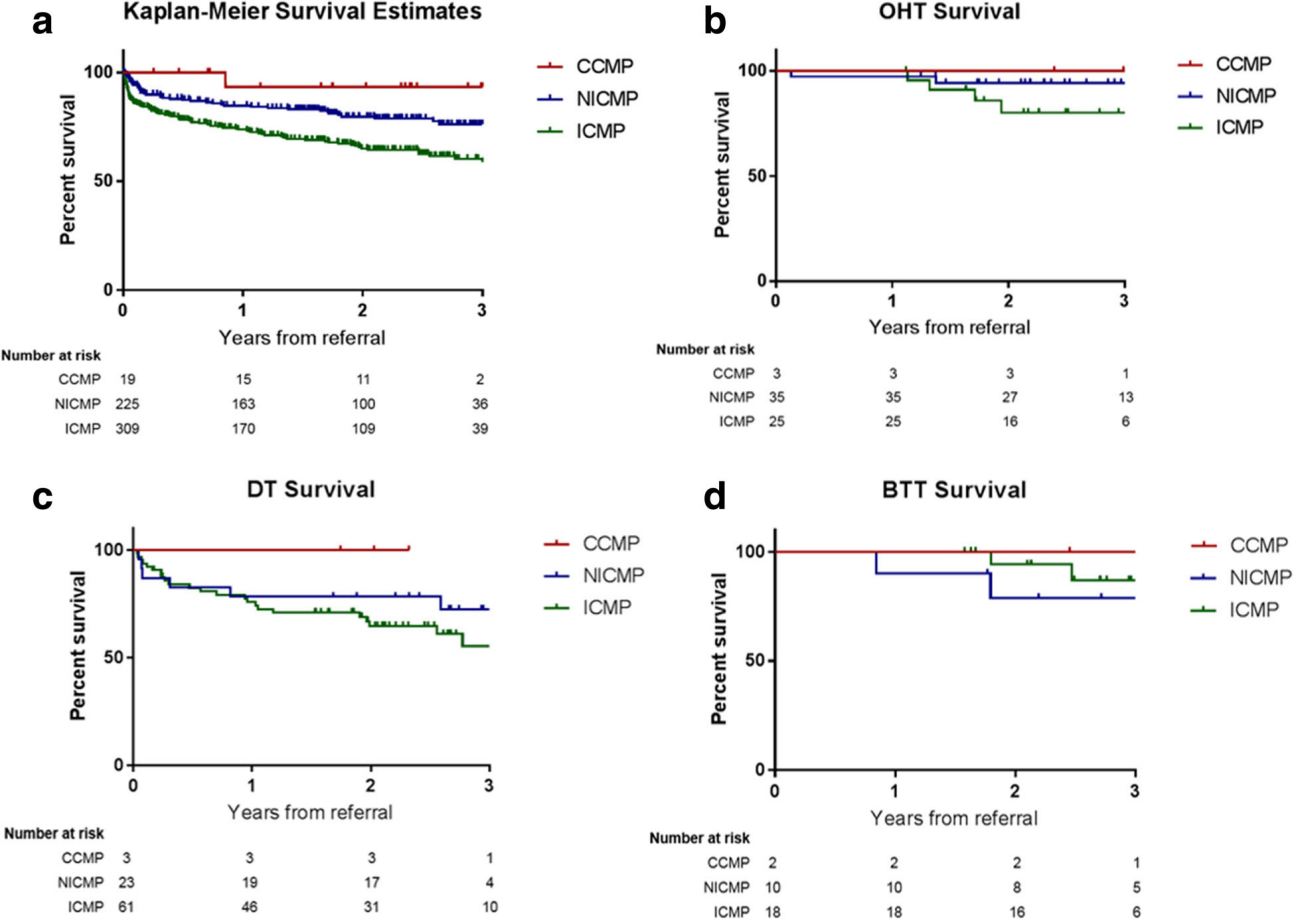
**a** Kaplan-Meier overall survival estimates from all groups. **b** Kaplan-Meier survival estimates in patients who underwent heart transplantation. **c** Kaplan-Meier survival estimates in patients who received an LVAD as DT. **d** Kaplan-Meier survival estimates in patients who received an LVAD as BTT

Overall short-term and long- term survival (1 year and 3 years) was analyzed (Fig. 1a); independent of type of advanced therapy, survival was significantly higher in the CCMP group when comparing CCMP vs NICMP vs ICMP (93.3% vs 84.8% vs 73.8%, respectively *P = 0.0021* for 1 year, 93.3% vs 76.2% vs 58.3%, respectively, *P = < 0.0001* for 3 year).

Consequently, we performed Kaplan-Meier survival analysis comparing patients whom underwent transplantation from each group (Fig. 1b), survival between patients who received LVAD as DT (Fig. 1c) and as BTT (Fig. 1d). For all analyses, the CCMP groups had better 3 year survival compared to the other groups; however, this did not reach statistical significance. When comparing survival for patients who did not undergo LVAD or OHT, CCMP 3-year survival was significantly higher compared to NICMP and ICMP (85.7%, 71.1%, and 53.6%, respectively, *p = 0.0013*). One year survival: 85.7% vs 82.4% vs 66.2%. (*p = 0.0012*) (not shown).

#### CCMP population characteristics

The incidence of CCMP was 3.4% among all cardiomyopathy patients, and CCMP accounted for 7.8% of non-ischemic CMP patients. The median follow-up was 740 days (range 93–1595). Average time from cancer diagnosis to referral was 16.8 years (± 9.6 years). Average time from referral to LVAD was 19.6 days (± 11.8 days). Average time from referral to OHT was 286.6 days (± 242.3 days). Average time alive after LVAD or OHT was 815.1 days (± 199.4 days) at the time analysis was performed, with 7/8 patients still alive. Average time from cancer diagnosis to advanced therapy 19.22 years (± 11.7 years). Table 2 describes demographic characteristics and diagnosis from CCMP patients. The mean age for the CCMP was 57.8 years, predominantly female (*n* = 13, 68%), and predominantly Caucasian (*n* = 9, 47%). Hypertension was the most common comorbidity in this group of patients (n = 13, 68.4%) followed by diabetes (*n* = 7, 36.8%) and obesity (*n* = 6, 31.6%). The principal cancer diagnosis was breast cancer (*n* = 8~ 42%), followed by hematologic cancers (n = 7~ 37%), 2 cases of Ewing Sarcoma (~ 10.5%), 1 bladder cancer case (~ 5%) and 1 lung cancer (~ 5%). Nine patients received both anthracycline-based chemotherapy plus radiation, 8 patients received anthracycline-based chemotherapy without radiation, and 2 patients received radiation therapy only (1 breast cancer, 1 lung cancer) (Table 2). Of note, the primary diagnosis of our CCMP population who received a transplant were Ewing sarcoma (*n* = 2) in childhood, breast cancer (*n* = 1), Hodgkin’s lymphoma (*n* = 1), and acute myeloid leukemia (*n* = 1). Baseline laboratory values and pulmonary function tests (PFTs) were collected before LVAD implant, OHT, and during first evaluations for patients who did not receive LVAD or OHT, however, there were no significant differences between these parameters.

**Table 2:** Study population characteristics (CCMP patients)

Total *n* = 19	
Age (yrs) *Mean (SD)*	57.8 ± 12.4
Male, *n (%)*	6 (31.6)
Race, *n (%)*	
Caucasian	9 (47.4)
Black	6 (31.6)
Hispanic	4 (21)
BMI *Mean (SD)*	28.5 ± 6.9
Comorbidities *n (%)*	
Diabetes	7 (36.8)
Hypertension	13 (68.4)
Hyperlipidemia	4 (21)
CAD	3 (15.8)
CKD	5 (26.3)
COPD	3 (15.8)
Tobacco	3 (15.8)
Obesity	6 (31.6)
Diagnosis, *n (%)*	
Breast Cancer	8 (42.1)
Hematologic	7 (36.8)
Non-Hodgkin Lymphoma	3 (42.9)
Hodgkin Lymphoma	2 (28.6)
DLBCL	1 (14.3)
AML	1 (14.3)
Other	4 (21)
Treatment	
Anthracycline-based chemotherapy	8 (42.1)
Radiation	2 (10.5)
Anthracycline-based chemotherapy plus radiation	9 (47.4)

From the 3 patients who received a DT LVAD, transplant was declined after medical review board due to age and comorbidities (mean age 76, range 72–84), although all 3 patients were in cancer remission. In the BTT group, time from LVAD implant to OHT was 695 days in 1 patient and 43 days in another patient. Overall, the waiting time from listing to transplant was 25.3 days in average (± 12.3).

Hemodynamic and echocardiographic parameters were analyzed between patients receiving LVAD as DT, BTT, transplanted patients, and patients whom received medical therapy only. Similarly, laboratory and pulmonary function values were compared between the same groups All parameters were analyzed before LVAD implant (for DT and BTT patients) and transplant, whereas parameters for patients who only had medical therapy were collected at the time of first evaluation. There were no statistically significant differences in hemodynamic, laboratory, or echocardiographic parameters between the groups (not shown).

##### MCS analysis

Demographic and clinical characteristics from all patients who underwent LVAD implantation were analyzed and compared according to etiology (CCMP vs NICMP vs ICMP). Patients who received an LVAD in the CCMP group were older (mean age 62.4 years vs 48.2 vs 60, *P value <  0.0001),* and predominantly Caucasian (60% vs 39% vs 43%, respectively, ns). Patients in the CCMP group had the lowest BMI at the time of implant compared to other etiologies (mean BMI 24.7 vs 31.9 and 28.2) and had fewer comorbidities. There were no differences in any hemodynamic parameters between the 3 groups. When analyzing echocardiographic measurements, ejection fraction (EF) was higher in the CCMP group (mean EF 23% vs 19.2% and 21.9%, P value 0.0085); Left Ventricular End Diastolic Diameter (LVIDd) and Left Ventricular End Systolic Diameter (LVIDs) were significantly lower in patients with CCMP than NICMP and ICMP patients (mean LVIDd 6.2 cm vs 7 vs 6.5, respectively, *P value 0.0168;* mean LVIDs 5.2 cm vs 6.3 cm vs 5.6 cm, respectively, *P Value 0.0032*). Mean fractional shortening (FS%) was significantly higher in patients with CCMP when compared to NICMP patients (15.8% vs 10.7%, respectively, *P* Value 0.0486). Patients in the CCMP group had less RV dysfunction pre-LVAD compared to other groups (by echo qualitative report). Similarly, 40 % of the patients on the CCMP group met criteria for right ventricular (RV) failure post-LVAD, compared to 18.2% from the NICMP group and 21.5% of the ICMP group. However, none of the patients on the CCMP were implanted with a right ventricular assist device (RVAD), compared to 12.1% of the NICMP and 13.9% of the ICMP. Of note, none of these differences were statistically significant (not shown). Patients in the CCMP group had significantly higher forced vital capacity (FVC) pre-LVAD compared to the NICMP and ICMP patients (4.6 vs 2.7 vs 2.8, respectively, *P value = 0.0212*) (not shown).

##### OHT analysis

Baseline demographic characteristics from patients who underwent heart transplantation (not including BTT patients), were analyzed. Patients in the ICMP group were significantly older when compared to other cardiomyopathies (mean 59.7, *P value 0.0447*). CCMP patients had a lower LVIDd compared to NICMP and ICMP patients (5.3 vs 5.7 vs 6.5, respectively, *P value 0.0415*) (not shown). International Normalized Ratio (INR) and prothrombin time (PT) were higher in the CCMP group (*p* = 0.0312, *p* = 0.0230, respectively) (not shown). Patients in the CCMP group had significantly lower forced expiratory volume (FEV_1_) compared to patients in NICMP and ICMP groups (1.4 vs 2.4 vs 2.2, respectively, *p* = 0.0276). Similarly, FVC was lower in the CCMO group (1.9 vs 3.2 vs 2.9, respectively *p* = 0.0341) (not shown).

## Discussion

The current study demonstrates that: 1) the incidence of cancer treatmentrelated cardiomyopathy is likely underreported in large retrospective databases and 2) patients with cancer treatment-related cardiomyopathy whom are referred for advanced therapies have non-inferior outcomes compared to patients with other cardiomyopathies.

In our cohort, in which retrospective chart review was meticulously performed to determine if patients met criteria for cancer treatment-related CMP, CCMP accounted for 3.4% of all referrals (and 7.8% of non-ischemic CMP referrals) for advanced heart failure therapies in a high volume institution This number is higher than previous estimates. This may be due to the use of a single center database in which the more specific etiology of dilated cardiomyopathy is able to be adjudicated more accurately. Importantly, our single-center study allows for precision in the diagnosis of CCMP and highlights that registry databases such as INTERMACS and UNOS may fail to identify these patients, instead labeling them inaccurately as “idiopathic” or “nonischemic”. Data submitted by our institution to UNOS and INTERMACS failed to correctly classify 60% and 40% of CCMP patients, respectively. Thus, to learn more about the specific population, we should ensure as a community that we put specific diagnoses into these databases. In addition, as the number of cardio-oncology and cancer survivorship programs continue to grow, it may be time to consider a registry of adult cancer survivors (in addition to existing pediatric registries such as the St.Judes Childhood Cancer Survivor Study) to accurately track long-term outcomes.

Another reasons for the high percentage of CCMP patients in our paper may be due to differences in the time periods of our studies and previous studies. Indeed, Lenneman et al. revealed that the percentage of CCMP undergoing heart transplantation annually had a significant increase since 1987, while the percentage of patients with idiopathic cardiomyopathy remained at a constant rate per year [10]. Additionally, the ISHLT registry data suggests that referrals might be increasing, exemplified by the fact that 23% of the transplants in the CCMP group occurred in the final year of the study (2000 to 2008), whereas transplants from NICMP patients remained at 11% per year [9]. This number is expected to increase as the number of cancer survivors continues to increase and the cardiotoxicities of novel therapies continue to be discovered. Novel agents such as tyrosine kinase inhibitors and immune checkpoint inhibitors, for example, have had a significantly greater incidence of cardiotoxicity in real-world data compared to initial studies [11, 12].

Additionally, one might postulate that the increased percentage of referrals compared to those that receive a transplant or LVAD reflect a higher likelihood to deny CCMP patients for advanced therapies. Our data, however, did not reflect that. In fact, a higher percentage of patients in the CCMP group received LVAD or OHT as advanced therapies compared to all the other patients.

Despite concerns of cancer recurrence and other non-cardiac adverse events related to cancer therapies, 11.6% of patients who underwent transplantation in our institution were CCMP cases, compared to 2.5% from ISHLT registry and to ~ 1% from UNOS database, reflecting that CCMP patients may be more common than previously thought and may also reflect imprecise diagnosis of CCMP, as discussed above [9, 10].

More than 4% (*n* = 5) of patients implanted with a durable MCS in our institution from patients referred between 2013 and 2016, were CCMP patients, compared to 2% of INTERMACS registry published in 2014. In the registry data, patients with CCMP (33%) were more likely to be implanted with the strategy of destination therapy compared to those with NICMP (14% implanted as DT), ICMP (23% as DT). Our database shows that implantation as BTT was at least equivalent in the CCMP group compared to other NICMP and ICMP groups (40% vs 34.3% vs 18.5%, respectively), although the total number of LVADs in CCMP patients in our cohort is small (*n* = 5).

Large registry data has shown that right ventricular dysfunction is more common in patients with CCMP undergoing LVAD implantation compared to other patients. For example, using INTERMACS registry data, [3] it was shown that the need for RVAD was twice as likely in patients with CCMP compared to patients with nonischemic cardiomyopathy (CCMP 19% vs ICMP 6% vs NICMP 11% [3]; and CCMP 5.6% vs NICMP 2.3% [10]). RV dysfunction was also more common in our CCMP patients compared to others, occurring in 40% (*n* = 2) of patients with CCMP compared to 18.2% (*n* = 6) and 21.5% (*n* = 17) of NICMP and ICMP, respectively. However, in contradistinction to previous studies, none of the patients with CCMP undergoing durable LVAD implantation required concomitant or subsequent RVAD implantation, compared to 12% (*n* = 4) of the NICMP and 14% (*n* = 11) of the ICMP patients [3]. This may be due to a small sample size or may be due to a decreased incidence of RVAD use in the present era, with an implantation rate that has decreased from 24.7% in 2006 to 5% in 2011 and 2.9% in 2012 [13]. However, if this decrease is due to better patient selection, in our cohort patients with CCMP referred for advanced therapies were less likely to be excluded from LVAD implantation than other patients.

It has been previously shown that CCMP patients treated with MCS had survival similar to that of ICM and other NICM patients (1-year, 2-year, and 3-year survival rates of 73%, 63%, and 47%, respectively) and no survival differences existed between the bridge-to-transplant (BTT) and DT cohorts [3]. However, our study shows that, although there was higher incidence of RV failure in the CCMP group, the CCMP patients have better survival than other NICMP and ICMP patients regardless of the type of therapy; LVAD, OHT, or medical therapy.

To our knowledge, no one has described outcomes of CCMP patients referred for advanced HF therapies without a bias towards the eventual outcome (i.e. transplant and LVAD registries). This is an important addition to the existing literature, showing that even outcomes of patients who are deferred or declined for LVAD and/or OHT have comparable outcomes compared to patients with other forms of cardiomyopathy. In addition, CCMP were equally eligible for advanced therapy options compared to patients with other forms of cardiomyopathy.

### Limitations

There are a number of limitations to our study. The small sample size of patients with CCMP, although higher than demonstrated before, the restriction to a single institution, and its retrospective nature. In addition, previous history of cancer and chemotherapy treatment and dosage in CCMP patients is limited to patient-reported history. Thus, the percent of patients reclassified from “idiopathic” or “NICMP” to CCMP shows the limitation of database- centered study and the need for a detailed and prospective database collection for future studies, and it also highlights the need for a careful history by treating cardiologists.

Another limitation of this study is that we predominantly have cardiomyopathy due to a specific class of chemotherapies (i.e. anthracyclines), with the exception of 2 patients with HF related to radiation therapy, thus, specific doses and type of anthracycline along with concomitant chemotherapies are not known for all patients. Many other types of potentially cardiotoxic chemotherapies are increasingly used and can cause cardiomyopathy, including her-2 antagonists, tyrosine-kinase inhibitors, and immunotherapies. The long-term implications of these agents on advanced heart failure outcomes remains to be studied.

Lastly, ischemic evaluations including coronary angiography data was not available for many of these patients due to the long time period between original diagnosis of cardiomyopathy and referral to our program; therefore, the possibility of concomitant ischemic heart disease cannot be completely excluded in some of the patients diagnosed as CCMP.

## Conclusions

In a single institution, CCMP accounts for approximately 3% of all referrals for advanced HF therapies. Contrary to concerns for previous cancer and sequelae of cancer treatment-related therapies excluding patients for advanced therapies, a higher percentage of CCMP underwent advanced HF therapies and with similar outcomes. In addition, survival was better in patients who did not receive either LVAD or OHT. Also, our study exemplifies that registry databases frequently fail to identify CCMP and labels these patients incorrectly as “idiopathic” or “nonischemic”. This is the first study to show that among patients referred for advanced therapies, CCMP patients do not have inferior outcomes compared to other cardiomyopathies regardless of the selected management strategy.

## Abbreviations

AML: Acute myeloid leukemia
BMI: Body mass index
BTT: Bridge to transplant
CAD: Coronary artery disease
CCMP: Cancer treatment-related cardiomyopathy
CKD: Chronic kidney disease
COPD: Chronic obstructive pulmonary disease
DLBCL: Diffuse large b-cell lymphoma
DT: Destination therapy
EF: Ejection fraction
FEV: Forced expiratory volume
FS: Fractional shortening
FVC: Forced vital capacity
HF: Heart failure
ICMP: Ischemic cardiomyopathy
INTERMACS: Interagency Registry for Mechanically Assisted Circulatory Support
IVSd: Interventricular septal thickness at diastole
LVAD: Left ventricular assist device
LVIDd: Left ventricular end-diastolic diameter
LVIDs: Left ventricular end-systolic diameter
LVPWD: Left ventricular posterior wall dimension
MCS: Mechanical circulatory support
NICMP: non-Ischemic cardiomyopathy
O-CMP: Other cardiomyopathies
OHT: Orthotropic heart transplant
PFTs: Pulmonary function tests
RV: Right ventricle
RVAD: Right ventricular assist device
UNOS: United Network for Organ Sharing
